# Criterion validation of two submaximal aerobic fitness tests, the self-monitoring Fox-walk test and the Åstrand cycle test in people with rheumatoid arthritis

**DOI:** 10.1186/1471-2474-15-305

**Published:** 2014-09-17

**Authors:** Birgitta Nordgren, Cecilia Fridén, Eva Jansson, Ted Österlund, Wilhelmus Johannes Grooten, Christina H Opava, Anette Rickenlund

**Affiliations:** Division of Physiotherapy, Department of Neurobiology, Care Sciences and Society, Karolinska Institutet, 23100, SE-141 83 Stockholm, Sweden; Division of Clinical Physiology C188, Department of Laboratory Medicine, Karolinska Institutet at Karolinska University Hospital, SE-141 86 Stockholm, Sweden; Department of Rheumatology, Karolinska University Hospital, SE-171 77 Stockholm, Sweden; Division of Clinical Physiology N101, Department of Molecular Medicine and Surgery, Karolinska Institutet at Karolinska University Hospital, SE-171 76 Stockholm, Sweden

**Keywords:** Aerobic capacity, Aerobic power, Exercise, Maximal oxygen uptake, Peak oxygen uptake

## Abstract

**Background:**

Aerobic capacity tests are important to evaluate exercise programs and to encourage individuals to have a physically active lifestyle. Submaximal tests, if proven valid and reliable could be used for estimation of maximal oxygen uptake (VO_2max_). The purpose of the study was to examine the criterion-validity of the submaximal self-monitoring Fox-walk test and the submaximal Åstrand cycle test against a maximal cycle test in people with rheumatoid arthritis (RA). A secondary aim was to study the influence of different formulas for age predicted maximal heart rate when estimating VO_2max_ by the Åstrand test.

**Methods:**

Twenty seven subjects (81% female), mean (SD) age 62 (8.1) years, diagnosed with RA since 17.9 (11.7) years, participated in the study. They performed the Fox-walk test (775 meters), the Åstrand test and the maximal cycle test (measured VO_2max_ test). Pearson’s correlation coefficients were calculated to determine the direction and strength of the association between the tests, and paired t-tests were used to test potential differences between the tests. Bland and Altman methods were used to assess whether there was any systematic disagreement between the submaximal tests and the maximal test.

**Results:**

The correlation between the estimated and measured VO_2max_ values were strong and ranged between r = 0.52 and r = 0.82 including the use of different formulas for age predicted maximal heart rate, when estimating VO_2max_ by the Åstrand test. VO_2max_ was overestimated by 30% by the Fox-walk test and underestimated by 10% by the Åstrand test corrected for age. When the different formulas for age predicted maximal heart rate were used, the results showed that two formulas better predicted maximal heart rate and consequently a more precise estimation of VO_2max_.

**Conclusions:**

Despite the fact that the Fox-walk test overestimated VO_2max_ substantially, the test is a promising method for self-monitoring VO_2max_ and further development of the test is encouraged. The Åstrand test should be considered as highly valid and feasible and the two newly developed formulas for predicting maximal heart rate according to age are preferable to use when estimating VO_2max_ by the Åstrand test.

**Electronic supplementary material:**

The online version of this article (doi:10.1186/1471-2474-15-305) contains supplementary material, which is available to authorized users.

## Background

Rheumatoid arthritis (RA) is a chronic inflammatory disease with primary symptoms of joint pain, fatigue and major impact on functioning and health. People with RA have an increased risk of cardiovascular events which might result from an interaction between traditional risk factors, those related to chronic inflammation and possibly to physical inactivity [1, 2].

Considering that physical activity (PA) is an important part of treatment and care in patients with RA, it is recommended that clinicians promote PA in this group [3]. In line with evidence based practice, the effectiveness of a period of PA should be assessed and evaluated. Since self-monitoring has been identified as an effective technique of increasing PA, clinicians should encourage patients to regularly self-monitor PA progress outside of the clinic [4]. The Fox-walk test is a novel method to estimate maximal oxygen uptake (VO_2max_) by walking on an outdoor track. It is easy to perform, self-administered and requires no expensive equipment. The Fox-walk test is highly reliable in people with RA with an intra class correlation (ICC) of 0.98 (95% Confidence Interval, CI: 0.95-0.99) and the reliability is not influenced by disease-related factors [5]. Moreover, the Fox-walk test is also a reliable method to monitor improvements in VO_2max_. On a group level, the smallest detectable differences should be an increase of >1 ml · kg^−1^ · min^−1^ (or 2.4%) to show a clinically relevant difference, whereas on an individual level, an increase of >2.8 ml · kg^−1^ · min^−1^ (or 9.4%) indicates a clinically relevant difference in VO_2max_[5]. However, the test still needs to be validated in people with RA.

Assessment of aerobic fitness usually takes place in a clinical setting and is commonly supervised by a health professional as a test leader. One of the most commonly used submaximal cycle ergometry tests, suitable for a clinical setting, is the Åstrand test [6]. The estimation of the VO_2max_ from the test is based on a linear relationship between mechanical load, oxygen uptake and heart rate (HR) obtained during the test. Although the test is recommended as an assessment method in physiotherapy guidelines in the management of patients with RA, it has not yet been tested for validity in this group [7].

If true maximal HR is not known, a prediction of the individual’s maximal HR is usually needed for estimation of VO_2max_ by the Åstrand test. To do this an age-correction factor was incorporated in 1960 to account for the decrease in maximal HR with age [8]. However, both this age correction factor and the most wide spread formula for age predicted maximal HR (220-age), developed in 1971 [9] may underestimate maximal HR in an elderly healthy population [10]. In order to increase the precision of the predicted maximal HR, and consequently the estimated VO_2max_, it has recently been suggested that the formula should be modified [10, 11]. Neither of these formulas have been evaluated in submaximal tests in people with RA.

## Purpose

The purpose of this study was to examine the criterion-validity of the submaximal self-monitoring Fox-walk test and the submaximal Åstrand test against a maximal cycle test in people with RA. A secondary aim was to study the influence of different formulas for age predicted maximal HR when estimating VO_2max_ by the Åstrand test.

## Methods

### Participants

Thirty participants diagnosed with RA according to the 1987 American College of Rheumatology criteria [12], aged 44–75 years, independent in daily living and with no Swedish language difficulties, were recruited from an ongoing PA trial, the PARA 2010 study (http://www.controlled-trials.com/ISRCTN25539102/). Participants were informed about the present study and signed consent was obtained. Three participants dropped out due to illness or personal reasons. Data on demographics, disease-related characteristics, medication and level of PA of the remaining 27 participants (22 females and 5 males) are displayed in Table 1. All subjects in this sample had been encouraged to exercise regularly the past year.

**Table 1:** **Characteristics of participants (n = 27)**

Characteristics	
Demographics	
Gender: female, n (%)	22 (81)
Age (yrs), mean (SD)	62 (8)
Anthropometrics	
Weight, kg mean (SD)	70 (14)
Height, cm, mean (SD)	170 (10)
BMI, mean (SD)	24.3 (3.5)
RA-related characteristics	
Disease duration (yrs), median (IQR)	15 (7–30)
DAS 28, median (IQR)^1^	2.4 (2.2-2.8)
General health (VAS), 0–100, median (IQR)	14 (7–25)
Fatigue (VAS), 0–100, median (IQR)	19 (6–35)
Pain (VAS), 0–100, median (IQR)	19 (7–28)
Activity limitation (HAQ) 0–3, median (IQR)	0.379 (0–0.750)
RA- medication^2^	
Biologics, n (%)	17 (63)
DMARD, n (%)	17 (63)
NSAID, n (%)	8 (30)
Corticosteroids, n (%)	5 (19)
Other medication	
Beta adrenergic antagonists, n (%)	3 (11)
Physical activity	
30 minutes moderate intensity, times/week past year, median(IQR) (n = 26)	3.1 (2.2-4.0)
Circuit training, times/week past year, median (IQR) (n = 26)	1.3 (0.7-1.7)

^1^n = 22, ^2^n = 25, SD = Standard deviation, BMI = Body mass index: weight (kg) / height (m^2^), DAS 28 = Disease activity score, VAS = visual analogue scale, HAQ = Health Assessment Questionnaire, IQR = Inter quartile range, DMARD = Disease modifying anti rheumatic disease, NSAID = Non steroidal anti inflammatory drug.

### Procedure

Participants were assessed with the submaximal Fox-walk test and the submaximal Åstrand test for estimation of VO_2max_ and a maximal exercise cycle test for direct measurement of VO_2max_ (measured VO_2max_ test). For practical reasons, the Fox-walk test was performed at least three days (at most five days) prior to or after the cycle tests. The submaximal Åstrand cycle test was performed on the same test occasion as the VO_2max_ test, separated by five minutes rest in between each test. The participants were informed to refrain from smoking and vigorous activity the day before the cycle test and from heavy meals within two hours before the test.

### Assessments

Demographics were collected with a self-administered questionnaire. General health perception [13], fatigue [14, 15] and pain [16] were rated on visual analogue scales (VAS, 0–100 mm), and activity limitation was assessed with the Stanford Health Assessment Questionnaire (HAQ) [17]. Data on disease duration, disease activity score (DAS 28) and medication was retrieved from patient files. Standing height was measured to the nearest 0.5 cm and body weight was measured with Tanita TBF-300 Body Composition Analyzer (Tanita Corporation of America, IInc. Illinois, USA). Body mass index (BMI) was calculated as weight in kilograms divided by the square of the height in metres and perceived exertion was rated on the Borg’s RPE scale [18].

#### Fox-walk test

The Fox-walk test tracks are situated on public places throughout Sweden and other European countries and consist of different lengths, ranging from 400 meters to 2500 meters. The test is performed walking or running, but only people with good aerobic fitness are recommended to do the test running. People have free access to the tracks. By recording the duration, time of walking or running and using this information, the result of the test can be obtained from a specific website (http://www.halsosparet.se/). The Fox-walk test was administered by two trained test leaders. The length of the track used in the present study was 775 meters and the height difference was two meters. The track was located centrally in Stockholm, Sweden. To get familiar with the track and as a warm up session, the test leaders walked the track together with the participants and explained the test procedure. Preceding the test, general health, fatigue and lower limb pain were rated and participants were instructed to walk the track with maximal effort without running. The test leaders recorded the time (Silva stopwatch, Sollentuna, Sweden) and collected data on perceived exertion with the Borg scale. Lower limb pain was rated again after the test was completed.

A previously developed equation, derived from a study with healthy people (unpublished observations) was used to estimate VO_2max_ using the Fox-walk test. Gender, age, height of the person, BMI, walking speed, length of the track as well as the track ascendance are entered in to the equation: 46.5 + 5.08 · sex (1 = men, 2 = women) -0.66 · age (years) -23.3 · height (meters)-0.388 · BMI (kg/m^2^) +24.95 · walking speed (m/s)-0.146 · total track ascendance (m). In the unpublished study the Fox-walk test was validated against laboratory tests of aerobic capacity on a cycle ergometer. The different variables in the equation were tested on their impact on the predictive value (sensitivity and specificity) with stepwise linear regressions and the equation was adjusted after these results.

#### Åstrand’s submaximal cycle test (Åstrand test)

An electrically braked cycle ergometer (Rodby, RE990, Rodby innovation AB, Uppsala, Sweden) with a 12-lead ECG (CASE/Carestream, GE Healthcare, Freiburg, Germany) was used. A starting load between 30–100 Watts (W) and an incremental mechanical load between 10–20 W/min was set individually for each participant depending on the predicted work capacity according to the standard reference values and the participant’s estimated fitness level [19]. A pedal frequency of 60 revolutions per minute was kept during the entire test. When the participant reached a HR exceeding 110 beats per minute and a rating of perceived exertion of 13 out of 20 according to the Borg’s RPE scale the ramp was ceased and work load was maintained for six minutes. HR was measured at the end of the fifth and sixth minutes of this stage, from which the mean HR was computed. The VO_2max_ was estimated using the Åstrand-Rhyming nomogram [8] based on mean HR at steady state and the mechanical load. With increasing age maximal HR decreases. The estimated VO_2max_ was therefore corrected for age. Alternatively, the estimated VO_2max_ was adjusted for maximal HR assessed at the maximal cycle test (measured VO_2max_ test).

To study the influence of other formulas for age predicted maximal HR to estimate VO_2max_ by the Åstrand test, the following formulas were used:

- the Fox-Haskell formula (220 - age) [9]
- the Tanaka formula (208–0.7 · age) [10]
- the Nes formula (211–0.64 · age) [11]

It should be noted that there is a similarity between names of the Fox-walk test and the Fox-Haskell formula (220-age) and these should not be mixed up.

#### Test for maximal oxygen uptake (measured VO_2max_ test)

Oxygen uptake and carbon dioxide elimination were measured during a ramp cycle ergometer test until volitional exhaustion. The same cycle ergometer cycle was used for the measured VO_2max_ test for the Åstrand test and the starting work load and ramp protocol was reset according to the previous Åstrand test. The participant was instructed to keep a cadence of 60 revolutions per minute until volitional exhaustion. Oxygen uptake and carbon dioxide elimination was measured by a breath-by-breath method, while the participant wore a Hans Rudolph mask (Vmax ENCORE 229, VIASYS™ Healthcare, Palm springs, CA/USA). Peak workload and HR were recorded as well as the peak oxygen uptake and carbon dioxide elimination averaged over a 20 second interval. True maximal workload and oxygen uptake require a high degree of engagement by both participant and staff, and are seldom reached in a laboratory setting. However, in line with most literature these variables are in the present study referred as maximal HR and VO_2max_. The test was accepted to be limited by the cardio respiratory system when the participants no longer could maintain the targeted 60 RPM along with at least two of the following criteria: a respiratory exchange ratio (RER) >1.10, rating of perceived exertion exceeded 16 out of 20 according to the Borg’s RPE scale and a maximal HR exceeding 90% of the estimated age-predicted maximal HR (220 - age) [9]. The measured VO_2max_ was determined from the highest 20-s period during the exercise before the test was interrupted. Before each test session the system were calibrated for respiratory gases and air flow using standardized gases and a 3 L calibration syringe, respectively.

### Data treatment and statistics

Descriptive data are presented as percentages, means (SD) and medians (IQR) when appropriate. To test the validity, Pearson’s correlation coefficients statistic were calculated to determine the direction and strength of the association between VO_2max_ estimated by the Fox-walk test and measured VO_2max_ test_,_ as well as between VO_2max_ estimated by the Åstrand test and measured VO_2max_ test. The correlation coefficient was interpreted according to Cohen (1988), whereby 0.10-0.29 was considered small, 0.30-0.49 was considered moderate and 0.50-1.0 was considered strong association [20]. Paired t-tests were used to test potential differences between these measures. This test was also used to calculate the potential differences in lower limb pain before and after the Fox-walk test. Bland and Altman methods were used to assess whether there was any systematic disagreement between the submaximal tests and the maximal test [21]. Calculations included the mean difference between the measures, the standard deviation of the differences (SD_difference_) and the 95% limits of agreement: mean ± 2 · SD_difference_. For all tests, the level of significance was set at ≤ 0.05. All analyses were performed using StatSoft™, STATISTICA, version10.0.

### Ethics approval

This study was approved, as part of the PARA 2010 study, by the Stockholm Regional Ethical Review Board (2011/1241-32).

## Results

All participants (n = 27) completed the Fox-walk test, the Åstrand test and the measured VO_2max_ test.

### Measured VO_2max_ test

The participants’ performance characteristics for the VO_2 max_ test are presented in Table 2. Twenty four of the 27 participants achieved a respiratory exchange ratio greater than 1.10. All participants reached a maximal HR close to or exceeding the estimated age-predicted maximal HR according to Fox-Haskell (220-age) and all except one participant rated their perceived exertion as 17 or more. The self-reported lower limb pain, median (IQR), was 9 (2–22) before and 10 (3–23) after the cycle test with no statistically significant difference between the ratings. Data of comparison between the measured VO_2max_ test, and the Fox-walk test and Åstrand tests, respectively, are shown in Table 3.

**Table 2:** **Data characteristics of measured VO**_**2max**_ **test (n = 27)**

Characteristics	Mean (SD)
RER at measured VO_2 max_	1.16 (0.08)
HR at rest (BPM)	72 (10)
HR at measured VO_2 max_ (BPM)	171 (10)
Percent of age-predicted max HR Fox-Haskell*	108 (6)
Percent of age-predicted max HR Tanaka**	104 (5)
Percent of age-predicted max HR Nes***	99 (5)
BP Systolic at rest (mmHg)	137 (18)
BP Diastolic at rest (mmHg)	85 (9)
BP Systolic at measured VO_2 max_(mmHg)	188 (20)
Maximal workload (Watt)	181 (61)
Measured VO_2 max_ (l/min)	
total group	2.38 (0.88)
female	2.81 (0.43)
male	4.54 (0.79)
Measured VO_2 max_ (ml · kg^−1^ · min^−1^)	
total group	33.2 (7.8)
female	31.6 (7.0)
male	40.0 (8,2)
Perceived exertion (Borg’s RPE scale 6–20)	18 (17–19)

Measured VO_2max_ = maximal oxygen uptake, RER = respiratory exchange ratio, HR = heart rate, BPM = beats per minute, *percent of age predicted maximal HR according to Fox-Haskell formula (220 – age), **according to Tanakas formula (208–0.7 · age), ***according to Nes’ formula (211–0.64 · age), BP = blood pressure, RPE = ratings of perceived exertion.

**Table 3:** **VO**_**2max**_ **values from the measured VO**_**2max**_ **test and the submaximal tests**

l/min	Mean (SD)	p-value	r-value^1^	p-value
Measured VO_2max_	2.4 (0.8)	-	-	-
Fox-walk test	3.2 (0.9)	**0.004**	0.81	**<0.001**
Åstrand test corrected for age	2.2 (0.6)	**0.039**	0.82	**<0.001**
Åstrand test corrected for maximal HR	2.4 (0.6)	0.956	0.82	**<0.001**
Fox-Haskell (220-age)	2.2 (0.6)	**0.037**	0.82	**<0.001**
Tanaka (208–0.7 · age)	2.3 (0.6)	0.289	0.81	**<0.001**
Nes (211–0.64 · age)	2.4 (0.7)	0.752	0.81	**<0.001**
**ml · kg** ^**1**^ **· min** ^**-1**^	**Mean (SD)**	**p-value**	**r-value**	**p-value**
Measured VO_2max_	33 (8)	-	-	-
Fox-walk test	44 (2)	**<0.001**	0.52	**0.006**
Åstrand test corrected for age	31 (8)	0.064	0.68	**<0.001**
Åstrand test corrected for maximal HR	34 (8)	0.593	0.65	**<0.001**
Fox-Haskell (220-age)	31 (8)	0.059	0.68	**<0.001**
Tanaka (208–0.7 · age)	32 (8)	0.546	0.66	**<0.001**
Nes (211–0.64 · age)	34 (8)	0.345	0.66	**<0.001**

Paired t-test comparisons between the measured VO_2max_ values and the six estimated VO_2 max_ values and Pearson’s correlation coefficient’s between the tests (n = 27).

^1^Pearson’s correlation coefficient, VO_2max_ = maximum oxygen uptake, HR = heart rate.

P≤0.05 in bold.

### Fox-walk test vs. measured VO_2max_ test

**Figure 1:**
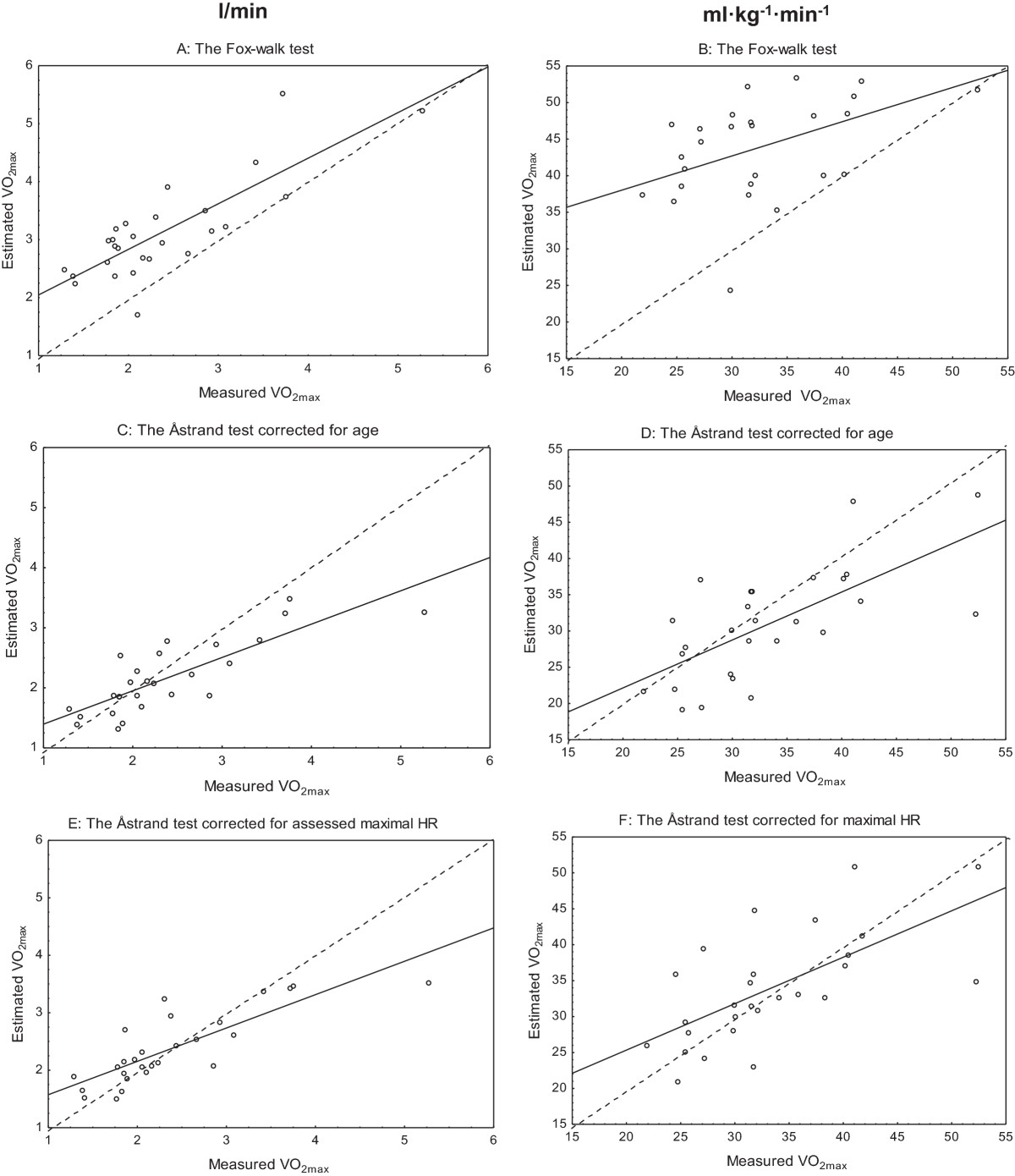
**Measured vs estimated maximal oxygen uptake (VO**_**2max**_ **).** Correlations between measured VO_2max_ and VO_2max_ estimated with the Fox-walk test **(A-B)**, expressed in l/min (left) and ml · kg^−1^ · min^−1^ (right). Correlations between measured VO_2max_ and VO_2max_ estimated with the Åstrand test corrected for age **(C-D)** and corrected for maximal HR **(E-F)**, expressed in l/min (left) and ml (right). The line of identity is plotted in the figures (n = 27).

**Figure 2:**
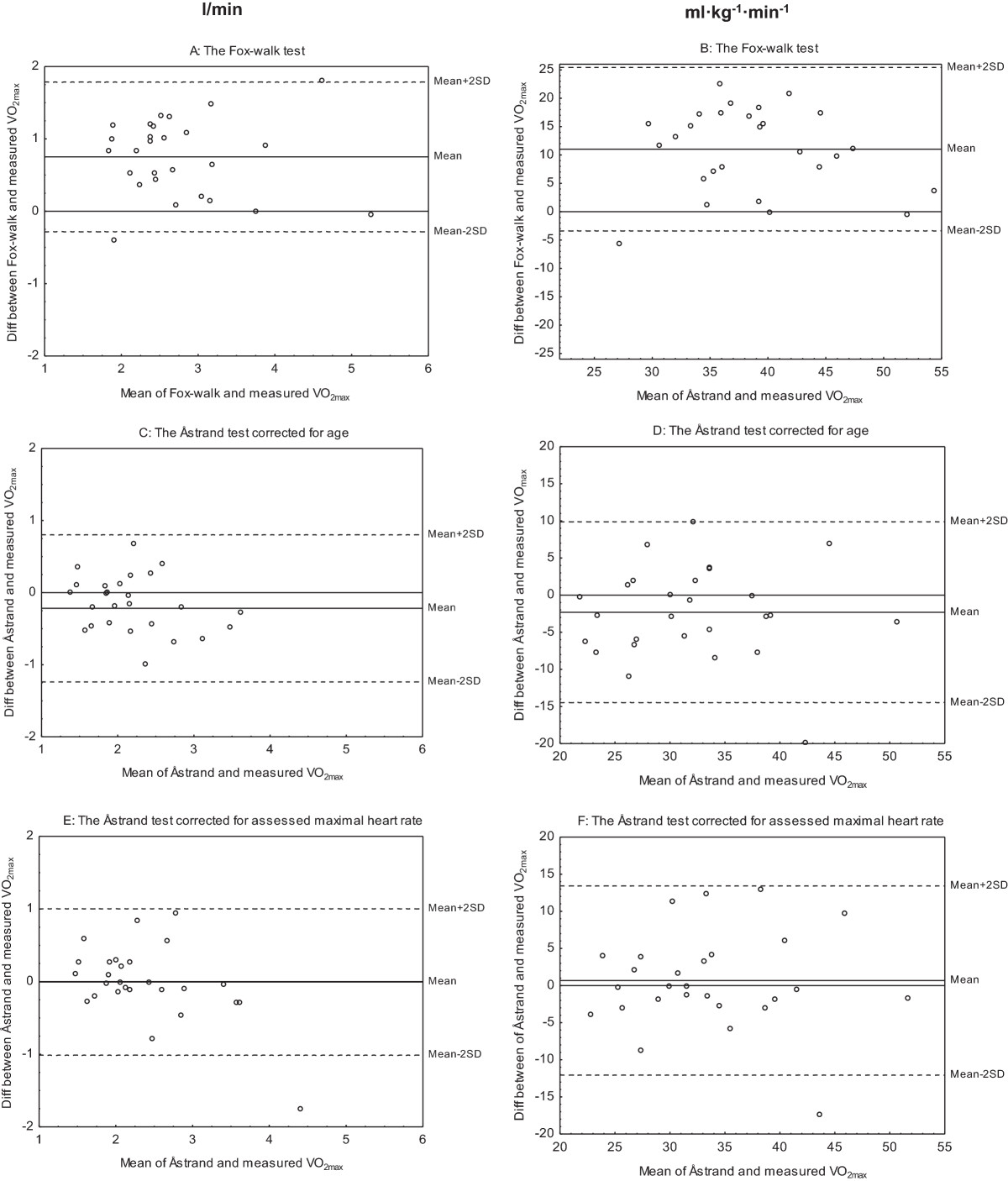
**Bland and Altman plots of measured and estimated maximal oxygen uptake (VO**_**2max**_ **).** Differences of VO_2max_ in l/min (left) and ml · kg^−1^ · min^−1^ (right) between the Fox-walk test and the measured VO_2max_ test, plotted against the mean of VO_2max_ of these two tests **(A-B)**. Differences of VO_2max_ in l/min (left) and ml · kg^−1^ · min^−1^ (right) between the Åstrand test and the measured VO_2max_ test, plotted against the mean of the VO_2max_ of these two tests. The estimated VO_2max_ with the Åstrand test is corrected for age **(C-D)** and assessed maximal heart rate **(E-F)**, respectively. The plotted lines in the figures show the 95% limits of agreement (n = 27).

Pearson’s correlation coefficients showed a strong, positive relationship between the estimated and measured VO_2max_ and ranged between r = 0.52 and r = 0.81 (Figure 1A-F and Table 3). The paired t-test revealed a significant difference between the tests, and the Fox-walk test overestimated VO_2max_ by almost 30% (Table 3). The Bland and Altman analyses showed that the distribution of differences of VO_2max_ was independent of VO_2max_ levels, regardless if it was expressed in l/min or adjusted for weight expressed in ml · kg^−1^ · min^−1^ (Figure 2A-F). The 95% limits of agreement for the estimated VO_2max_ were wide, ranging from −0.3 to 1.8 l/min and −3.4 to 25.4 ml · kg^−1^ · min^−1^, respectively. The self-reported lower limb pain was, median (IQR), 19 (7–28) before and 21 (8–39) after the Fox-walk test with no statistically significant differences between the ratings. Perceived exertion was, median (IQR), 15 (9–17) after the Fox-walk test.

### Åstrand test corrected for age vs. measured VO_2max_ test

Pearson’s correlation coefficients showed a strong and positive relationship between the estimated and measured VO_2max_ and ranged between r = 0.68 and r = 0.82 (Figure 1A-F and Table 3). The paired t-test revealed a significant difference between the tests, and the Åstrand test underestimated VO_2max_ by almost 10% (Table 3). The Bland and Altman analyses showed that the distribution of differences of VO_2max_ was independent of VO_2max_ levels, regardless if it was expressed in l/min or adjusted for weight expressed in ml · kg^−1^ · min^−1^, respectively (Figure 2A-F). The 95% limits of agreement ranged from −1.2 to 0.8 l/min and −14.4 to 9.8 ml · kg^−1^ · min^−1^, respectively and in most cases differences between measures were less than 0.6 l/min and 8 ml · kg^−1^ · min^−1^, respectively.

### Åstrand test corrected for assessed maximal HR vs. measured VO_2max_ test

Pearson’s correlation coefficients showed a strong, positive relationship between the estimated and measured VO_2max_ and ranged between r = 0.65 and r = 0.82 (Figure 1A-F and Table 3). The paired t-test did not reveal a significant difference between the tests (Table 3). The Bland and Altman analysis demonstrated good agreement between the two tests, and no systematic over- or underestimation were present (Figure 2A-F). The 95% limits of agreement ranged from −1 to 1 l/min and −12.1 to 13.4 ml · kg^−1^ · min^−1^. Several cases were distributed around zero and in most cases the differences between the measures were less than 0.3 l/min and 8 ml · kg^−1^ · min^−1^.

### Åstrand test corrected for age predicted maximal HR with different formulas vs. measured VO_2max_ test

**Figure 3:**
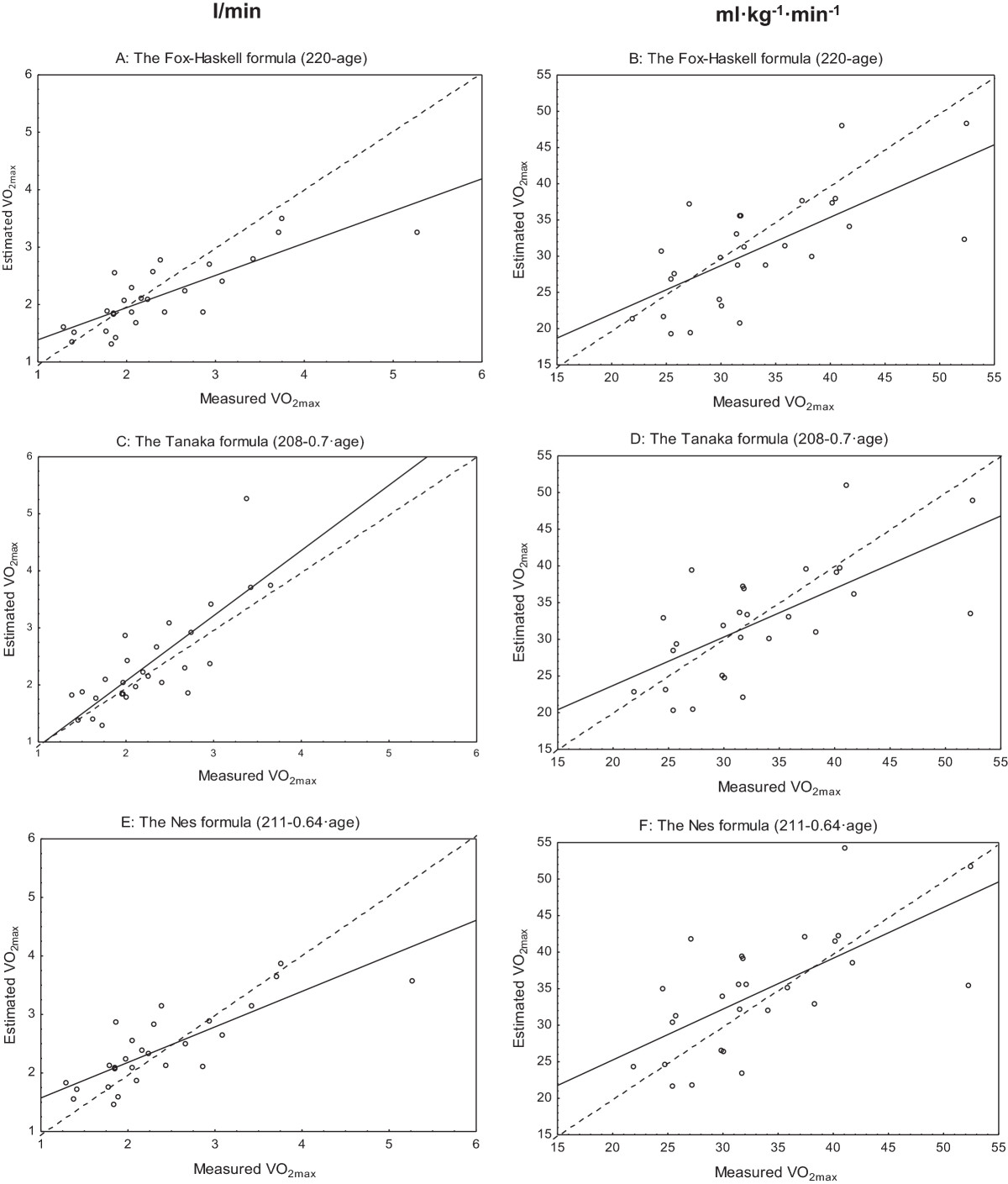
**Measured vs estimated maximal oxygen uptake (VO**_**2max**_ **).** Correlations between measured VO_2max_ and VO_2max_ estimated with the Åstrand test using age predicted maximal HR corrections with the Fox-Haskell **(A-B)**, the Tanaka **(C-D)** and the Nes **(E-F)** formulas, respectively, expressed in l/min (left) and ml · kg^−1^ · min^−1^ (right). The line of identity is plotted in the figures (n = 27).

**Figure 4:**
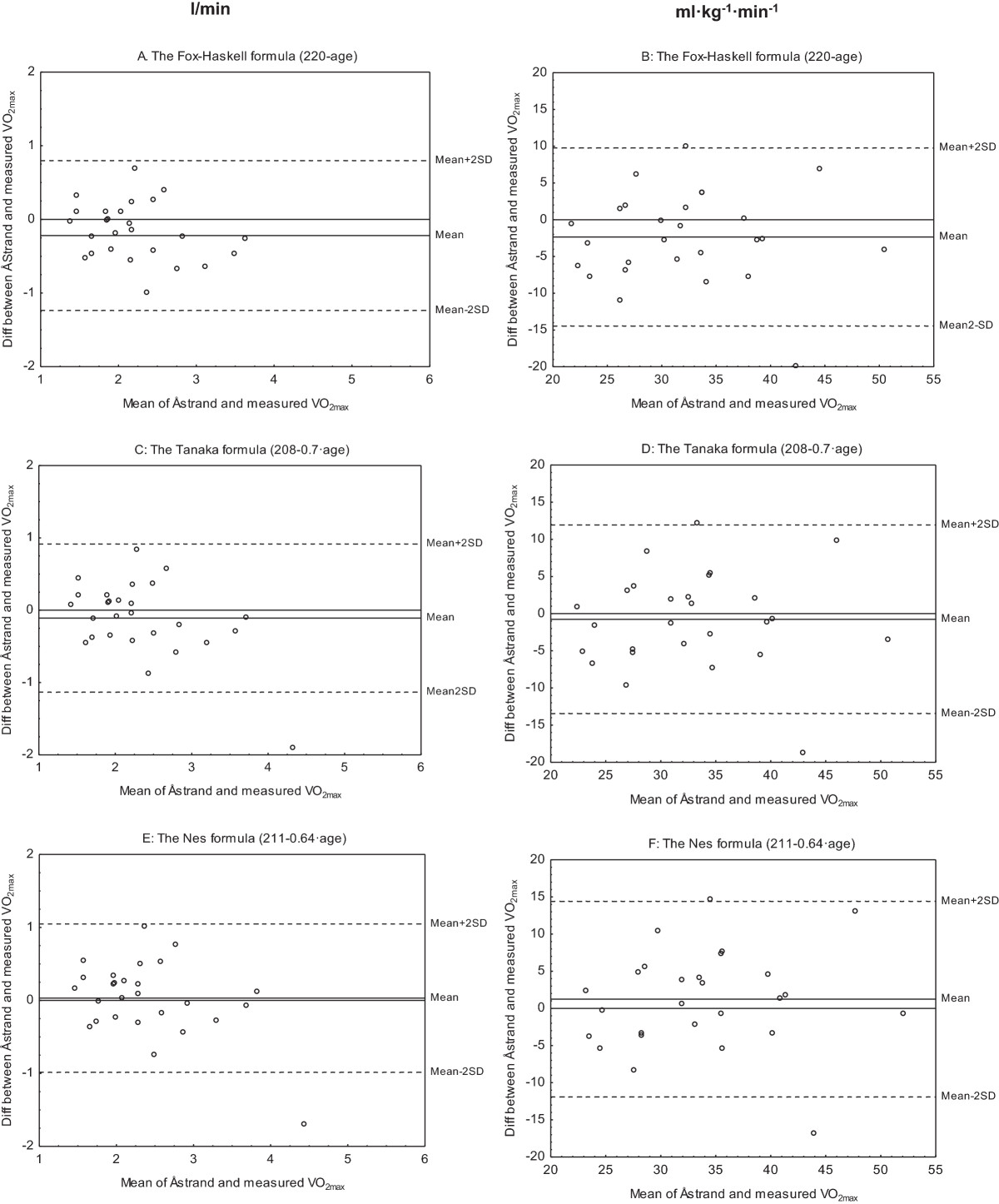
**Bland and Altman plots of estimated and measured maximal oxygen uptake (VO**_**2max**_ **).** Differences of VO_2max_ in l/min (left) and ml · kg^1^ · min^−1^ (right) between Åstrand test and the measured VO_2max_ test plotted against the mean of these two tests. The estimated VO_2max_ with the Åstrand test is corrected for age predicted maximal heart rate with the Fox-Haskell **(A-B)**, the Tanaka **(C-D)** and the Nes **(E-F)** formulas, respectively. The plotted lines in the figures show the 95% limits of agreement (n = 27).

Pearson’s correlation coefficients showed a strong, positive relationship between estimated and measured VO_2max_ and ranged between r = 0.66 and r = 0.82 (Figure 3A-F and Table 3). The paired t-test combined with the Bland and Altman analysis showed that maximal HR correction according to the Fox-Haskell formula (220-age) underestimated VO_2max_ expressed in l/min (Figure 4A-F and Table 3). No statistically significant underestimation was found when maximal HR was corrected according to the Tanaka (208–0.7 · age) or Nes (211–0.64 · age) formulas, or for values expressed in l/min or for values expressed in ml · kg^−1^ · min^−1^ (Figure 4A-F and Table 3).

## Discussion

This is the first study to examine the criterion validity of the submaximal Fox-walk test, a self-monitoring test aiming to estimate VO_2max_. The results showed that the test overestimated VO_2 max_ substantially, which should be taken into account when interpreting the results. However, despite this limitation the test could be useful for self-monitoring of aerobic fitness. To the best of our knowledge, this is also the only study to date which has examined the criterion validity of the submaximal Åstrand test in a population with RA and the test is considered to be a valid instrument to estimate VO_2max_ in physically active people with RA.

The Fox-walk test overestimated VO_2max_ by almost 30%, independent of participants’ levels of fitness. The overestimation could be explained by several factors. Some participants rated a low perceived exertion (five rated lower than 13) indicating that they should have performed the test running, as recommended for individuals with a high VO_2max_. However, this could consequently have lead to an underestimation of VO_2max_ and not an overestimation, as was the case with the Fox-walk test. Pain in lower limbs is likely to affect performance in a population with RA and could have had an impact on the test results. This was probably not a limiting factor for the participants in the present study, indicated by the low rating of lower limb pain after walking the track and it is therefore unlikely that this could have influenced the associations between the two methods. Another factor explaining the discrepancy between the two methods could have been that the measured VO_2max_ test was not performed with maximal exhaustion. However, a majority of the participants met the criteria for a maximal test and therefore a systematic interruption of the test at a submaximal level of exhaustion is unlikely and could not explain the large difference between the Fox walk test and the measured VO_2 max_ test.

The submaximal Åstrand test showed a strong correlation with the VO_2max_ test when corrected for age expressed in l/min (r = 0.82) but weaker correlation when expressed in ml · kg^-1·^min^−1^ (r = 0.68). The slightly lower relative (ml · kg^−1^ · min^−1^ ) compared to the absolute (l/min) value should be regarded as a mathematic consequence of weight index giving a lower range in relation to the mean and thereby less good prerequisites for getting high r-values. In the present study, the Åstrand test underestimated VO_2max_ by 10%, which is in accordance with previous studies on healthy individuals [22, 23], although an overestimation also has been shown [24]. The assumption of a linear relationship between heart rate and VO_2max_, makes the estimation of VO_2max_ from a submaximal test strongly dependent on the accuracy of the age-predicted maximal HR. Tanaka’s (208–0.7 · age) [10] and Nes (211–0.64 · age) [11] formulas turned out to better predict maximal HR compared to the Fox-Haskell formula (220-age) [9], (99%, 104% and 108%, respectively, of assessed maximal HR). When age-correction was made with the use of these three alternative age-predicted HR max formulas, the widespread Fox-Haskell formula underestimated VO_2max_ by the same degree as Åstrand corrected for age [8, 25], whereas the two formulas by Tanaka and Nes seem to come closer to the measured VO_2max_ .

Some limitations associated with this study need to be considered. The population in the present study participated in an intervention promoting physical activity and they had exercised regularly during the past year, and were well-trained. Additionally the participants in this study had low disease-activity compared to people with RA in general. In addition, a majority of the individuals included in the present study were females which also could have hampered the generalizability of the results. Three subjects used low-dose beta-adrenergic antagonists for treatment of hypertension. This could have influenced the study results according to beta blockers side-effects on HR response. However, all subjects in this study reached a maximal HR between 98% and 120% (median 107%) of the estimated age-predicted maximal HR. According to the normal HR response in these subjects, use of β-blocker antihypertensive treatment had no or limited effects on HR response in relation to work load. When performing a cycle test for the first time, anxiety and inexperience with the test situation could have an impact on the test result. The work efficiency could be lower and the ratio between the HR and the work load could be higher, consequently leading to an underestimation of VO_2max_. However, this was probably not the case in this study as all participants had performed the test at least twice and were familiar with exercise testing. A strength in our study was that the same biomedical scientist (T Ö) conducted all cycle ergometry tests. With regards to the Fox-walk test, the test was performed on a single track and no other tracks were tested, thus future studies should consider that different results may be obtained on other tracks.

## Conclusions

The Fox-walk test cannot be used confidently for estimating VO_2max_ on the bases of the correlation and agreement analyses. However, the test may still be used but with consideration of its limitations when interpreting the results. We strongly recommend and encourage further development of the test, since it is a promising test for self-monitoring VO_2max_ by individuals outside of a clinical setting, and could also be used by professionals in the clinic. Provided that the Åstrand test is standardized according to the test manual, it should be considered as highly valid and feasible [26] in physically active people with RA and is recommended for use by health professionals in both clinical and research settings. The newly developed formulas by Tanaka and Nes for predicting maximal heart rate according to age are preferable [10, 11], but the Åstrand test is still valid with the use of its own age prediction VO_2max_ or with the Fox-Haskell formula for predicting maximal heart rate.

## Authors’ original submitted files for images

Below are the links to the authors’ original submitted files for images. Authors’ original file for figure 1 Authors’ original file for figure 2 Authors’ original file for figure 3 Authors’ original file for figure 4

## Abbreviations

BMI: Body mass index
DAS 28: Disease activity score
HAQ: Stanford Health Assessment Questionnaire
HR: Heart rate
PA: Physical activity
PARA: Physical activity in rheumatoid arthritis
RA: Rheumatoid arthritis
VAS: Visual analogue scale
VO_2max_: Maximal oxygen uptake
W: Watt.
