# Supplementary material for: Criterion validation of two submaximal aerobic fitness tests, the self-monitoring Fox-walk test and the Åstrand cycle test in people with rheumatoid arthritis
Source: BMC Musculoskelet Disord. 2014 Sep 17;15:305. doi: 10.1186/1471-2474-15-305 (PMC4180316; doi:10.1186/1471-2474-15-305)
Supplement: Supplementary file 1 — Authors’ original file for figure 1 [file 12891_2014_2247_MOESM1_ESM.pdf]

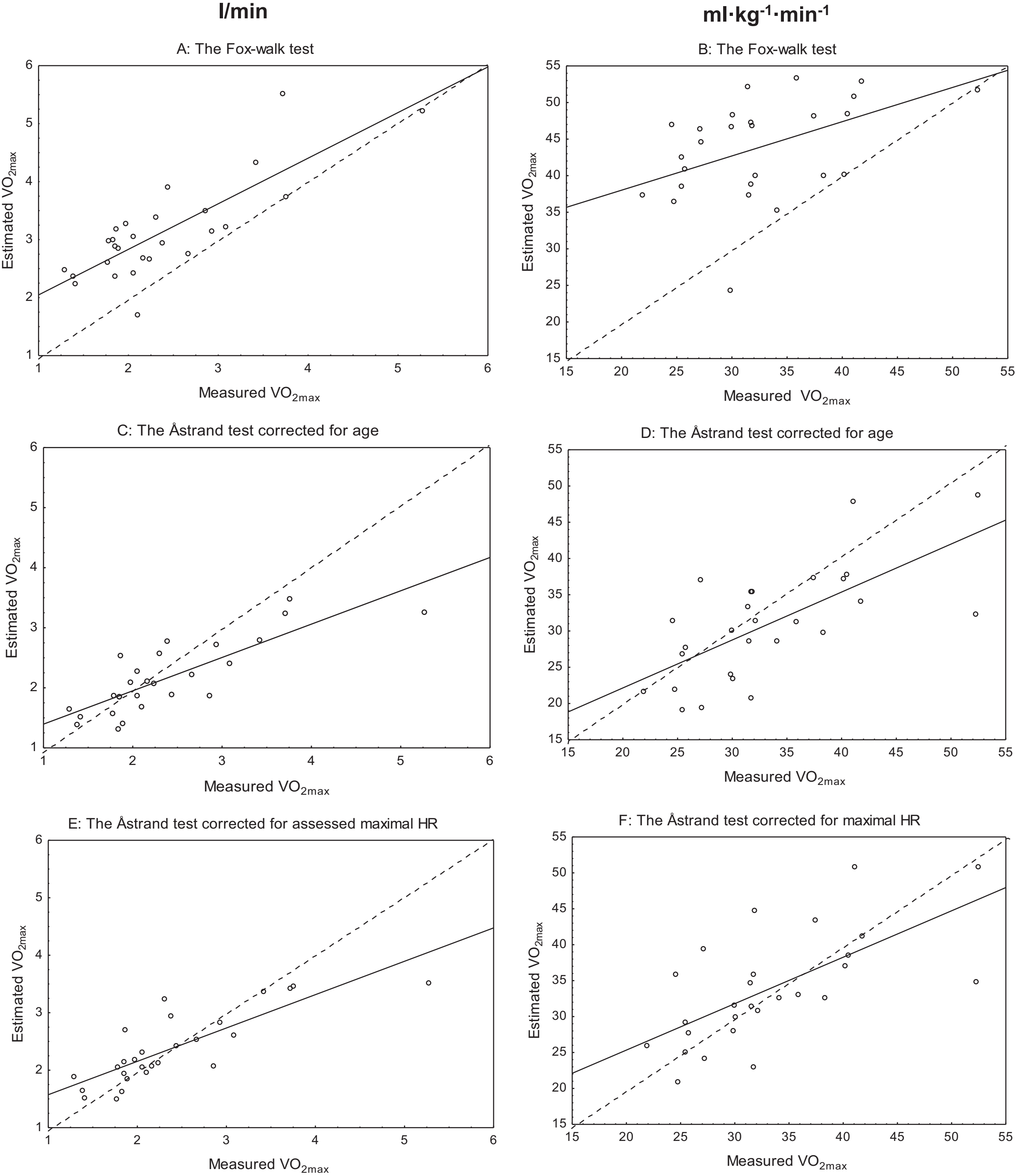
l/min
ml·kg-1·min-1
A: The Fox-walk test
B: The Fox-walk test
C: The Åstrand test corrected for age
D: The Åstrand test corrected for age
E: The Åstrand test corrected for assessed maximal HR
F: The Åstrand test corrected for maximal HR
Estimated VO2max
Measured VO2max
