# Supplementary material for: Criterion validation of two submaximal aerobic fitness tests, the self-monitoring Fox-walk test and the Åstrand cycle test in people with rheumatoid arthritis
Source: BMC Musculoskelet Disord. 2014 Sep 17;15:305. doi: 10.1186/1471-2474-15-305 (PMC4180316; doi:10.1186/1471-2474-15-305)
Supplement: Supplementary file 2 — Authors’ original file for figure 2 [file 12891_2014_2247_MOESM2_ESM.pdf]

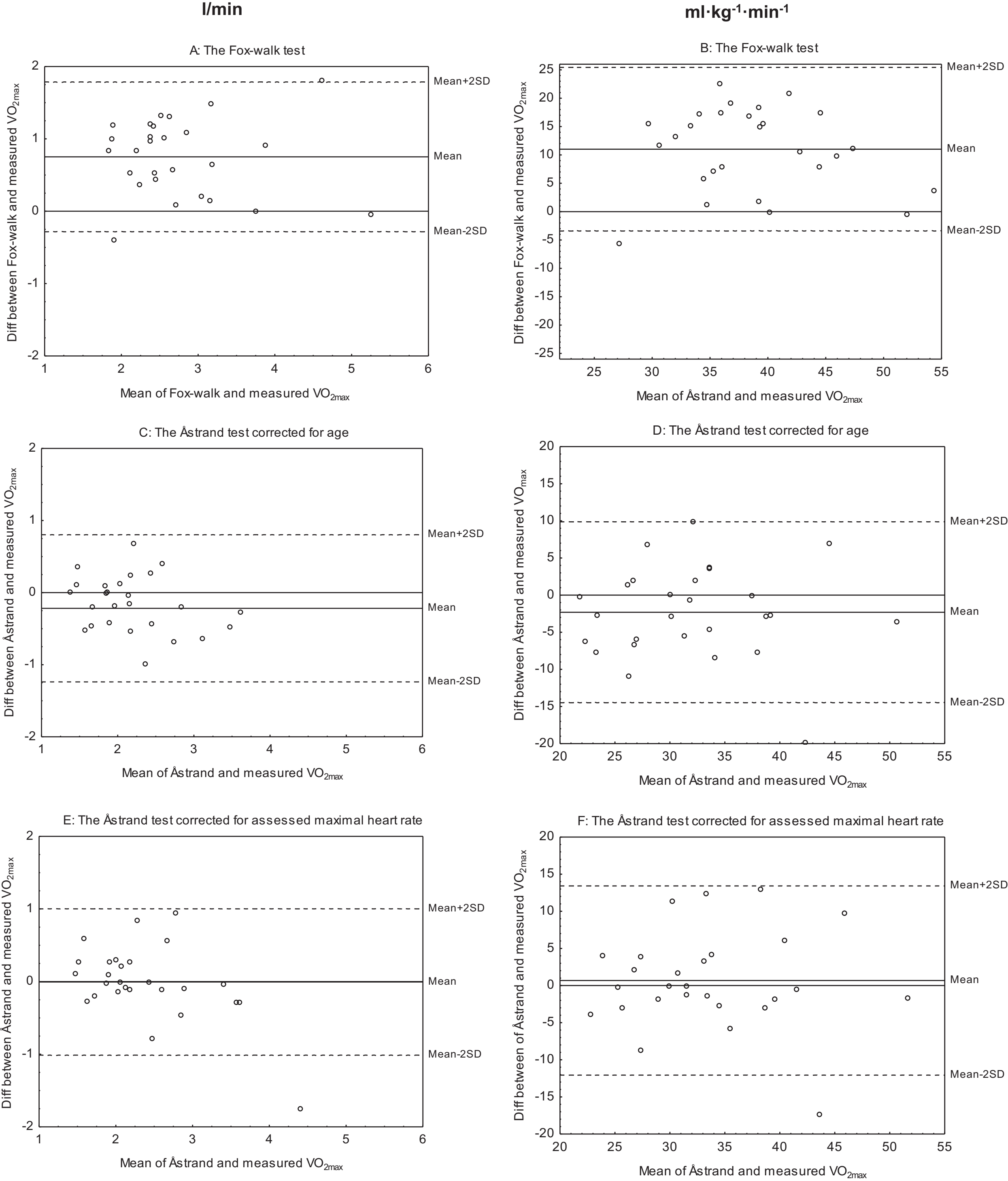
l/min
ml·kg-1·min-1
A: The Fox-walk test
Diff between Fox-walk and measured VO2max
Mean of Fox-walk and measured VO2max
Mean+2SD
Mean
Mean-2SD
B: The Fox-walk test
Diff between Fox-walk and measured VO2max
Mean of Åstrand and measured VO2max
C: The Åstrand test corrected for age
Diff between Åstrand and measured VO2max
Mean of Åstrand and measured VO2max
D: The Åstrand test corrected for age
Diff between Åstrand and measured VOmax
Mean of Åstrand and measured VO2max
E: The Åstrand test corrected for assessed maximal heart rate
Diff between Åstrand and measured VO2max
Mean of Åstrand and measured VO2max
F: The Åstrand test corrected for assessed maximal heart rate
Diff between of Åstrand and measured VO2max
Mean of Åstrand and measured VO2max
