# Supplementary material for: Criterion validation of two submaximal aerobic fitness tests, the self-monitoring Fox-walk test and the Åstrand cycle test in people with rheumatoid arthritis
Source: BMC Musculoskelet Disord. 2014 Sep 17;15:305. doi: 10.1186/1471-2474-15-305 (PMC4180316; doi:10.1186/1471-2474-15-305)
Supplement: Supplementary file 4 — Authors’ original file for figure 4 [file 12891_2014_2247_MOESM4_ESM.pdf]

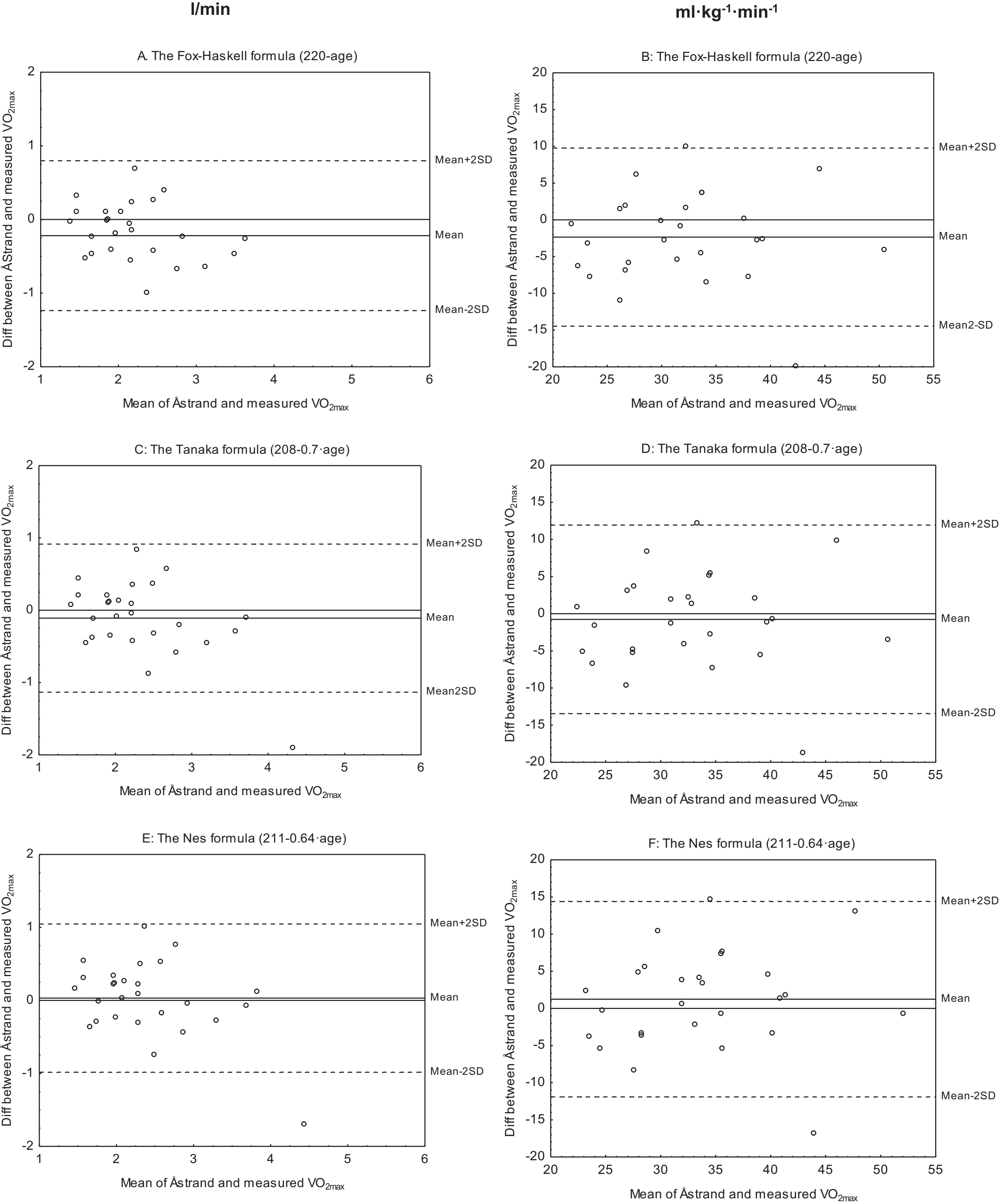
l/min
ml·kg-1·min-1
A. The Fox-Haskell formula (220-age)
B: The Fox-Haskell formula (220-age)
C: The Tanaka formula (208-0.7·age)
D: The Tanaka formula (208-0.7·age)
E: The Nes formula (211-0.64·age)
F: The Nes formula (211-0.64·age)
Diff between Åstrand and measured VO2max
Mean of Åstrand and measured VO2max
Mean+2SD
Mean
Mean-2SD
Mean2-SD
Mean2SD
